# Three-Dimensional Comparison of CBCT and Intraoral Scans for Assessing Orthodontic Traction of Impacted Canines with Clear Aligners

**DOI:** 10.3390/dj13070286

**Published:** 2025-06-24

**Authors:** Teresa Pinho, João Pedro Carvalho

**Affiliations:** 1 UNIPRO—Oral Pathology and Rehabilitation Research Unit, University Institute of Health Science (IUCS), CESPU, 4585-116 Gandra, Portugal; a29614@alunos.cespu.pt; 2 UMIB—Multidisciplinary Biomedical Research Unit, Abel Salazar Institute of Biomedical Sciences (ICBAS), University of Porto, 4050-313 Porto, Portugal

**Keywords:** cuspid, tooth, impacted, cone-beam computed tomography, 3D imaging, clear aligners

## Abstract

**Background**: Canine impaction complicates treatment and prolongs duration, requiring precise localization. CBCT is the gold standard for diagnosis and assessment. However, it involves high radiation exposure and cost. This study aimed to evaluate the effectiveness of a combined biomechanical approach for orthodontic traction of impacted maxillary canines (IMCs) and to determine whether intraoral scans (STL files) could replace a final CBCT in assessing canine repositioning. **Methods**: The sample included 10 patients (7 males and 3 females) with 13 severely displaced IMCs, treated with a protocol combining Invisalign^®^ aligners, elastics, mini-implants, and sectional wires. In all, 9 IMC were palatally impacted, while 4 were buccally impacted. A representative clinical case is presented to illustrate the biomechanics used in one of the complex cases. Canine movement was evaluated at the cusp and apex through two methods: overlay of pre- and post-treatment CBCTs, and overlay of initial and final STL scans onto the initial CBCT. **Results**: A Class I canine relationship was successfully achieved in all patients. No statistically significant differences were found between the two measurement methods (*p* > 0.05). **Conclusions**: Orthodontic traction of IMC, especially in complex cases, can be achieved using aligners, elastics, mini-implants, and sectional wires. Once the canine crown has erupted and is clinically visible, STL scans overlaid with the initial CBCT can accurately assess the final position of the crown and root. This allows clinicians to avoid a second CBCT in selected cases, reducing patient radiation exposure while maintaining diagnostic accuracy.

## 1. Introduction

Impacted maxillary canines (IMCs) are the most frequently impacted teeth following third molars, with an incidence ranging from 1.7% to 4.7% in the general population [1,2]. IMC impaction is more common in women than in men and occurs more often unilaterally than bilaterally [3,4]. According to Bishara et al. [3], the incidence of bilateral impaction in IMC patients is 8%. In two-thirds of cases, the palatine zone is affected, while only one-third involves the vestibular zone. Additionally, mandibular canine impaction is significantly less frequent compared to maxillary canines [3].

The etiology of maxillary canine impaction remains uncertain, but several factors influence it. Two theories explain its cause. The guidance theory suggests that erupting canines lack an orientation guide, mainly provided by the lateral incisor’s distal root surface [5]. In contrast, the genetic theory attributes impacted canines (ICs) to heredity, poor dental germ position, and developmental complications. The ‘sequential hypothesis’ suggests these theories may act at different stages of maxillary canine development [6].

Treating impacted teeth extends the duration and complexity of overall orthodontic treatment [7]. A precise diagnosis requires a comprehensive clinical and radiographic evaluation of the IC, including its location in 2D or 3D, while considering the challenges of orthodontic traction [8,9].

Accurate localization of IMCs is crucial and primarily depends on radiographic imaging. Orthopantomography (OPG) is the most commonly used initial diagnostic tool, offering two-dimensional information about the canine position [10].

Early evaluation of the impacted canine’s location on an OPG can serve as a useful predictor of treatment outcomes. However, compared to traditional 2D methods such as OPG, which has limitations, particularly in accurately determining the exact bucco-palatal position of the impacted tooth, 3D imaging techniques offer a more comprehensive view of the spatial relationships between structures. They overcome common drawbacks of 2D imaging, such as distortion, magnification errors, image artifacts, and overlapping structures, enabling a more accurate diagnosis [11,12].

Cone-beam computed tomography (CBCT) is the preferred imaging method for diagnosing impacted teeth and planning appropriate treatment. Accurate 3D position of the IMC is vital in clinical practice for evaluating its location, inclination, relationship to surrounding structures, bone coverage, and potential effects on adjacent teeth, such as root resorption [13,14].

However, this type of scan has some limitations, such as the high cost and the patient’s exposure to radiation. In addition, children are more radiosensitive than adults and face a higher risk of adverse effects from ionizing radiation [15,16]. Following the ALARA principle, healthcare workers must ensure the benefits of radiation outweigh the risks and minimize exposure below dose limits. Thus, the use of CBCT should only be used when clinically justified [17].

Despite the growing clinical use of intraoral STL scans, there is a lack of studies that directly compare their diagnostic reliability with CBCT in assessing both crown and root positioning in impacted maxillary canines. This gap is clinically significant, as CBCT involves radiation exposure and cost, particularly concerning in young patients. Assessing whether STL can replace a second CBCT may reduce exposure without compromising accuracy.

This study proposes a novel hybrid method, overlaying STL scans with the initial CBCT, to track canine movement. Unlike previous research that examines each method separately, we evaluated their combined use to determine the final tooth position. This approach may offer a safer, more efficient protocol for clinical follow-up.

The main objectives of this study are to evaluate the effectiveness of elastics, mini-implants, sectional wire, and aligners to orthodontic traction IMC, verifying the position of the canine and its movement during orthodontic treatment. This study also aimed to investigate whether intraoral scanners can replace the final CBCT by assessing the canine eruption position by overlaying the scan with the initial CBCT, eliminating the need for a final CBCT. By overlaying STL intraoral scans with the initial CBCT, we aimed to track tooth movement and assess final canine positioning without requiring a second CBCT. This method uniquely combines the detailed anatomical context of CBCT with the radiation-free, cost-effective advantages of STL scanning. Therefore, we defined the following null hypotheses: H0: There are no statistically significant differences between canine displacement measurements obtained from the final CBCT and those obtained by overlaying intraoral scans with the initial CBCT.

## 2. Data Description

### 2.1. Study Design

This study aimed to determine whether intraoral scanners can replace the final CBCT by assessing the canine position. Therefore, both the initial CBCT and the post-treatment CBCT scans of these patients were superimposed and compared to measure the movement of the canine cusp and apex. The same measurements were performed using the initial CBCT and intraoral scans (STL format), and the results from both methods were compared. A quantitative, comparative, and observational longitudinal cohort study was conducted.

### 2.2. Samples and Eligibility Criteria

Considering the inclusion and exclusion criteria, the study involved 10 patients (13 IMC) with ages ranging from 11 to 15 at the time of treatment. They underwent orthodontic treatment with elastics, mini-implants, sectional wires, and Invisalign® aligners (Porto, Portugal) at Clínica Manuel Neves, Lda in Porto, and at Clínica Médico-Dentária de São João da Madeira, Lda, Portugal. All treatments were performed by Dr. Teresa Pinho, a Specialist in Orthodontics and Invisalign® Diamond Provider.

Participants were eligible for inclusion in this study only if they met the following criteria:

- Patients who had at least one IC in a difficult position at the beginning of treatment (T0), requiring the use of traction methods for its eruption.
- Patients who underwent a CBCT before and, in some cases, at the end of traction, as well as STL files taken before and after.
- Patients who underwent orthodontic treatment with clear aligners and whose ICs were tractioned by auxiliaries like elastics, mini-implants, and sectional wire.

Patients who did not meet these criteria were excluded. Furthermore, all patients in the study provided informed consent upon entering the clinic, agreeing that their photographic and radiographic records, gathered for diagnostic and clinical monitoring, could be used in research work, articles, and scientific presentations. Their confidentiality was always guaranteed.

This research is part of a dissertation project that, as it involved data from patients from a private clinic, required approval from the Ethics Committee of the University Institute of Health Sciences (CESPU), with reference 38/CE-IUCS/2024.

### 2.3. Data Collection Procedures

The following data were collected: personal information (age and gender), the orthodontic method used (clear aligners, elastics, mini-implants, and sectional wires), the vestibular-palatal position of the IMC, treatment duration, intraoral photographs, initial and final intraoral scans, and radiographic records, including the initial CBCT. When available, the CBCT taken near or at the end of treatment was also collected.

CBCT scans and STL files were collected for measurement purposes. As the cases were treated with Invisalign^®^ aligners, arch scans were recorded and saved as STL files. The STL files from the beginning of treatment (T0) and the final stage of treatment (TF) were collected.

CBCT scans were performed before the start of treatment and, in some cases, after the complete crown eruption of the canines into the arch, although not necessarily at the end of the full treatment. These post-eruption CBCTs were used to verify the position of the canine and adjacent teeth, and were performed simultaneously with an STL scan, just before requesting additional aligners. After treatment completion, a final STL scan was obtained for all cases.

Data collection instruments:

- CBCT scans;
- Intraoral scans (STL files);
- Photographic records;
- Orthodontic and clinical patient reports.

Data processing instruments:

- Blue Sky Plan^®^ 4.12.13;
- RealGUIDE 5.3^®^;
- Microsoft Excel version 2016, 16.0.5448.1000;
- IBM^®^ SPSS^®^ Software version 29.0.

### 2.4. Assessment of Canine Impaction Difficulty Using CBCT-Based Index

A ‘difficult position’ is defined as a canine requiring surgical exposure due to its ectopic location, unfavorable angulation, or significant vertical and/or horizontal displacement, making spontaneous eruption unlikely. According to the study by Saleh et al., the severity of impacted maxillary canines increased when the cusp tips were closer to the midline and the root apices were farther away. Additionally, a greater vertical distance from the maxillary occlusal plane and a larger angulation of the IC was associated with increased treatment difficulty [18].

The difficulty index proposed by Chauhan et al. was used to evaluate impacted maxillary canines based on cone-beam computed tomography (CBCT) scans. This index scores multiple variables, including angulation, vertical position, bucco-palatal position, horizontal position, and rotation, and categorizes the cases into three levels of difficulty. Patients were classified as having minimum difficulty if the score was 5, moderate difficulty if the score was 6–10, and maximum difficulty if the score was 11–15 [19].

### 2.5. Measurement Procedures: CBCT Method

**First step: Segmentation of Teeth and Skull Bones from Initial CBCT.**

Each CBCT scan was opened in the Blue Sky Plan 4^®^ software. The CBCT was automatically segmented to isolate the skull bones from the upper teeth, creating a skull model. The maxillary bone was used as a reference structure. To generate a precise model of the IC, including the cusp tip and apex, automatic tooth segmentation was performed. The axial view was used to verify whether the cusp tip and apex were accurately included in the model. In some cases, manual segmentation was necessary to ensure their integration. The final model combined both the skull and the IC. This process was carried out only when both (initial and after canine crown eruption) CBCT scans of a patient were available.

**Second step: Overlay the Two CBCTs.**

The two models were superimposed and aligned using at least ten points located on the skull and then saved as an STL file.

**Third step: Measurements.**

The STL file was then imported into the RealGUIDE 5.3^®^ software. Three-dimensional measurements were taken between the cusp tips of the superimposed canines to assess their displacement, with the same measurements applied to the apex. By analyzing the treatment duration and the distance the canine moved at both the cusp and apex, the displacement rate (mm/month) was calculated for each site.

### 2.6. Measurement Procedures: Initial CBCT and STL Overlay to Evaluate Canine Displacement

**Figure 1:**
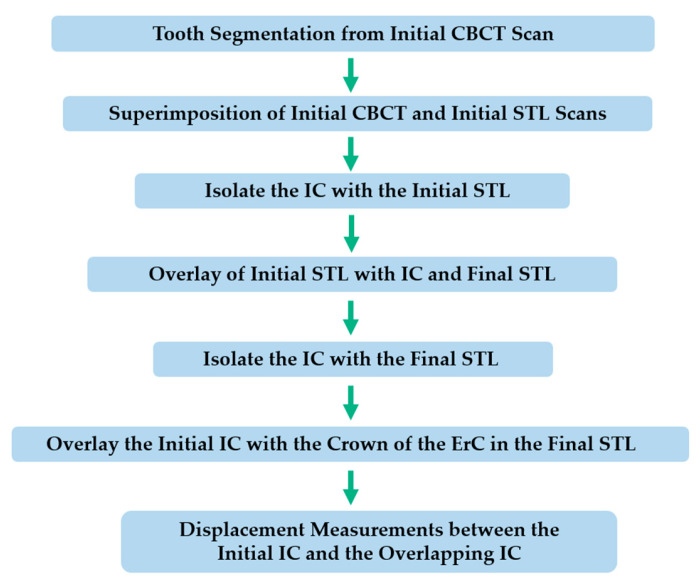
Step-by-Step protocol for 3D evaluation of canine position using the STL overlay method. Abbreviations: ErC: Erupted Canine; IC: Impacted Canine.

Out of the total sample, 8 cases had both STL scans and final CBCT available for comparison. In some cases, performing a CBCT scan at the end of treatment was not justified. Then, the possibility of measuring canine displacement at the tip and apex using another method was evaluated. This procedure was divided into five main steps, as described below. A diagram was created to better illustrate the sequence of the method (Figure 1).

**First step: Tooth Segmentation from Initial CBCT Scan.**

**Figure 2:**
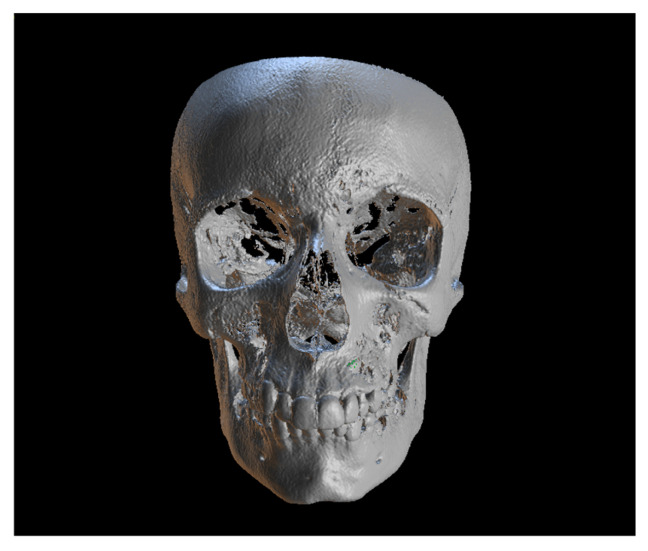
Initial CBCT scan.

**Figure 3:**
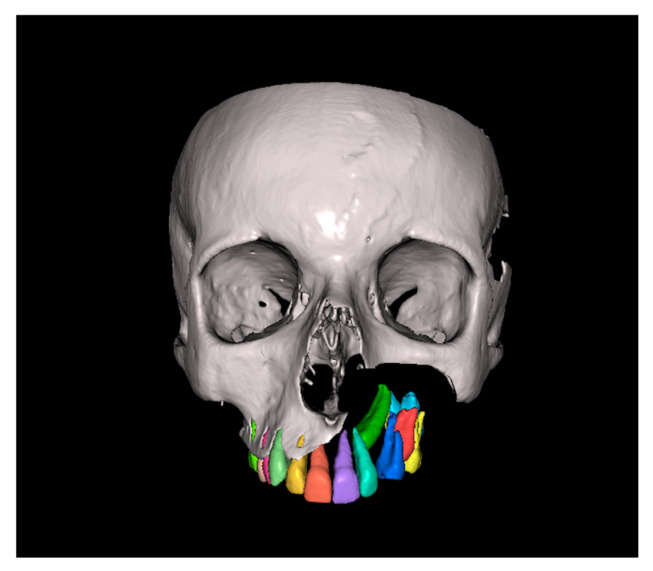
Initial CBCT after tooth segmentation.

Since the IC is not visible in the initial STL, it was necessary to know its three-dimensional location. As performed in the other method, the initial CBCT scan (Figure 2) was opened in the program, and all teeth were segmented. Although Figure 2 shows the skull model, only the teeth segmentation is necessary for this method. A small cut was made to allow visualization of the IC (Figure 3).

**Second step: Superimposition of Initial CBCT and Initial STL Scans.**

**Figure 4:**
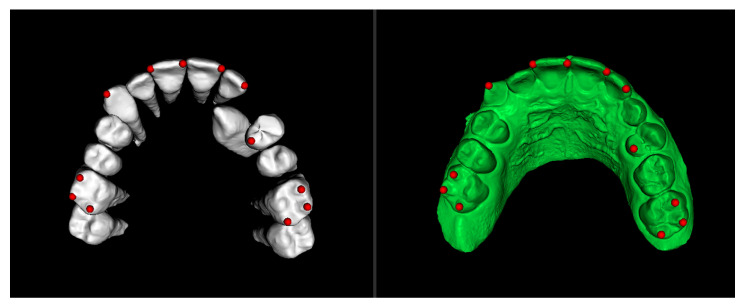
Superimposition of segmented teeth from initial CBCT and initial STL.

In this step, the segmented teeth from the initial CBCT scan were superimposed onto the initial STL file. This was achieved by marking corresponding points on several teeth throughout the dental arch, ensuring accurate alignment (Figure 4). Despite the morphological differences between the CBCT and STL models, particularly related to the canine region, as the permanent canine was still impacted and therefore visible only in the CBCT, corresponding anatomical landmarks were carefully selected on both models to enable accurate superimposition and comparison. The deciduous canine is only visible in the intraoral scanner data, as it was not segmented and included in this figure despite being present in the CBCT (Figure 4).

**Figure 5:**
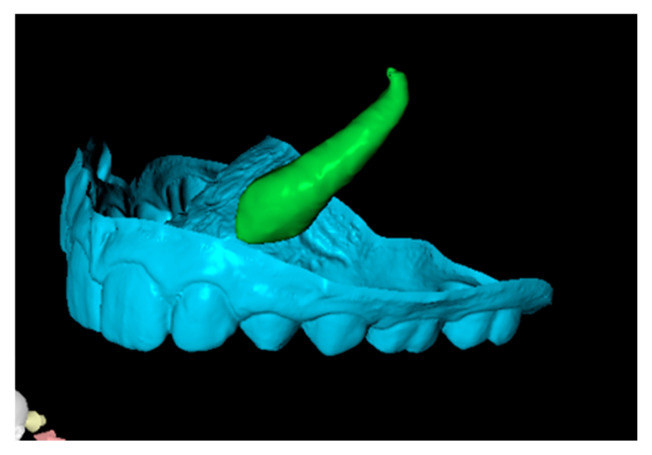
Initial STL with the IC from the CBCT scan.

Since orthodontic traction had not yet started, the position of the teeth remained unchanged between scans, allowing precise registration. As a result, the initial STL was effectively combined with the three-dimensional position of the IC derived from the CBCT (Figure 5).

**Third step: Overlay of Initial STL with IC and Final STL.**

**Figure 6:**
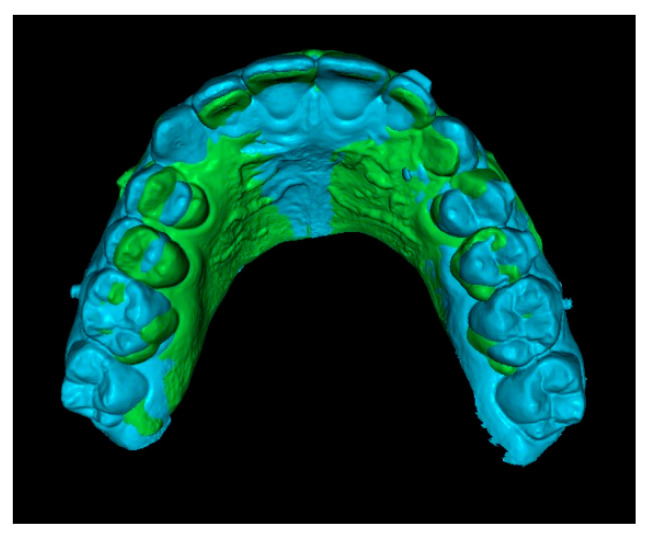
Superimposition of initial and final STL models using palatal rugae.

**Figure 7:**
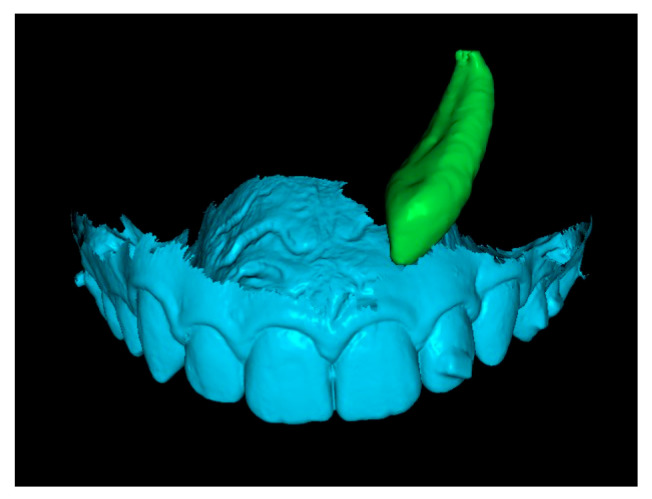
Final STL including the initial 3D position of the IC.

The initial STL combined with the IC was superimposed onto the final STL by marking stable reference points on the palatal rugae (Figure 6). This process resulted in a final STL model that includes the initial position of the IC (Figure 7).

**Fourth step: Overlap the Initial Canine with the Crown of the Canine in the Final STL.**

**Figure 8:**
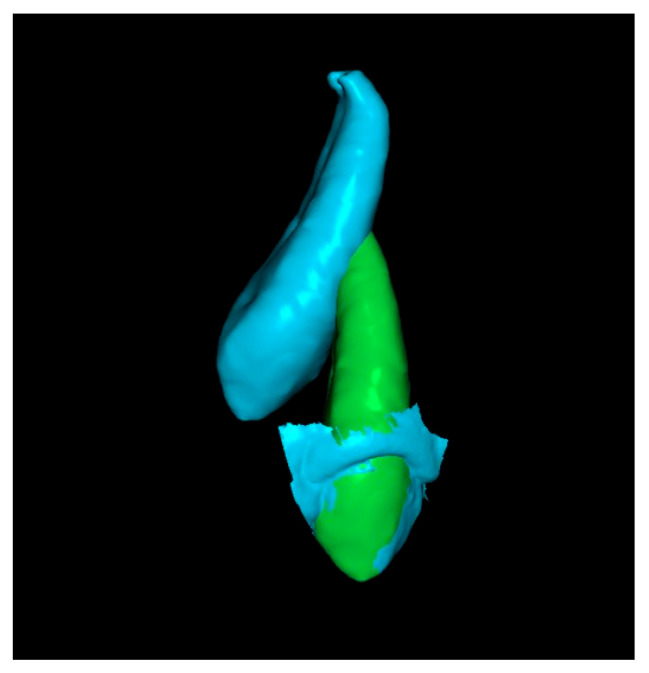
Overlay of canine in initial and final positions.

Since the final STL does not include tooth roots, only the crowns of the initial and final canines were overlapped. This alignment positioned the canine in its final position after traction. In some cases, after overlapping, the apex of the canine was not visible (Figure 8). When this occurred, a small cut was made to enable visualization of the apex.

**Fifth step: Displacement Measurements.**

**Figure 9:**
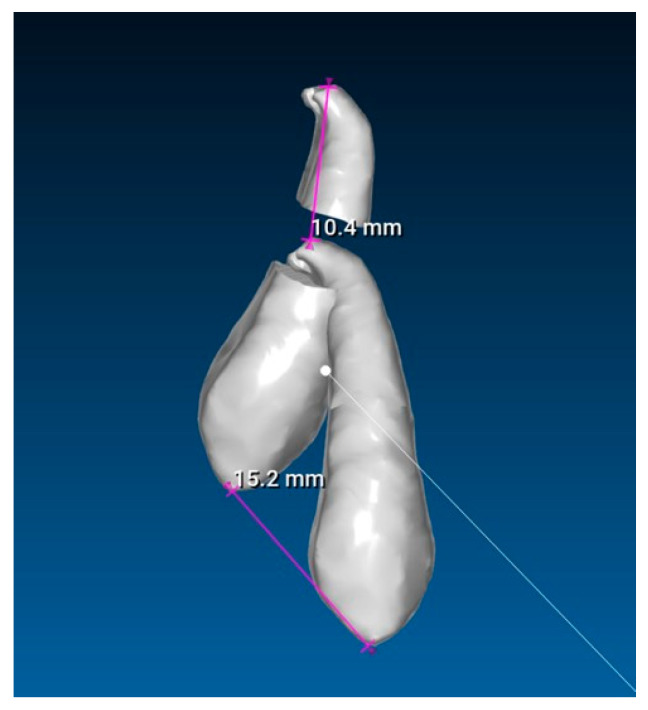
Cusp and apex displacement measurements.

The previous models of the initial and final canines were isolated and saved as separate STL files. Displacement measurements of the cusp and apex were then performed using the RealGUIDE 5.3^®^ software (Figure 9). All evaluations and measurements were performed in a blinded manner, with the primary evaluator unaware of patient identities throughout the study. The entire assessment process was conducted by a single author and was continuously supervised and reviewed by a second author. A high level of agreement was consistently observed, supporting the reliability of the methodology.

### 2.7. Statistical Analysis

Data analysis was performed using the IBM^®^ SPSS^®^ (Statistical Program for Social Sciences) software, version 29.0 for Windows. Descriptive statistics were produced, providing estimates of frequencies and percentages, means, medians, standard deviations, minimums, and maximums. The Shapiro–Wilk test was used to assess the normality of the sample, with no evidence of rejection of the null hypotheses. The normality of the data led us to adopt the paired *t*-test to compare the measurements of the canine displacement obtained from CBCT scans and intraoral scanners. To measure the magnitude of the effect, Cohen’s d proved to be the most appropriate measure, and, in this sense, the following guidelines were respected: |d| ≤ 0.20 seen as a small effect, |d| = 0.50 as a moderate effect and |d| ≥ 0.80 as a large effect [20]. The significance level was set at 0.05.

## 3. Results

### 3.1. Characteristics of the Clinical Study Sample

**Figure 10:**
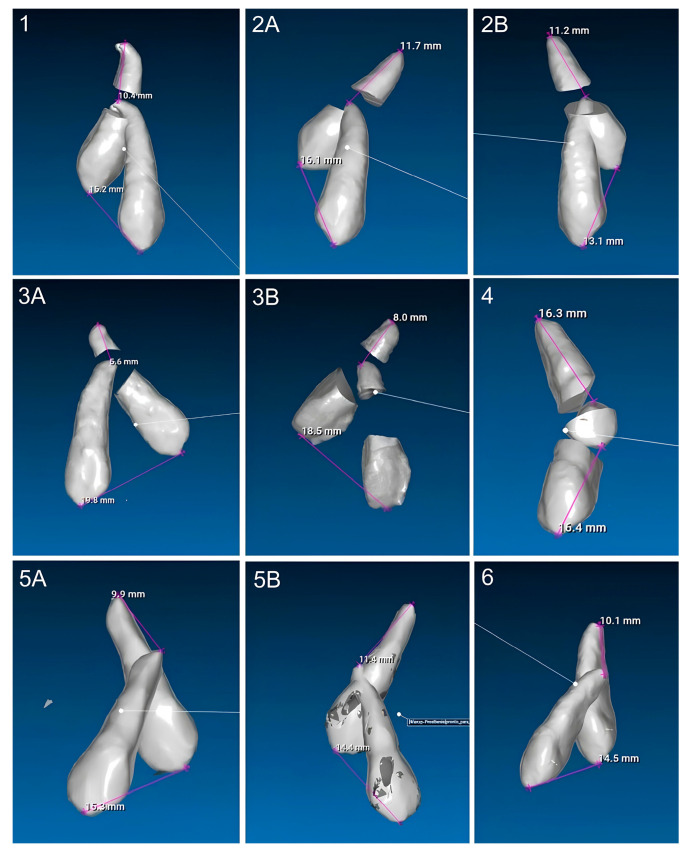
Measurements of all palatally ICs in the sample (n = 9).

**Figure 11:**
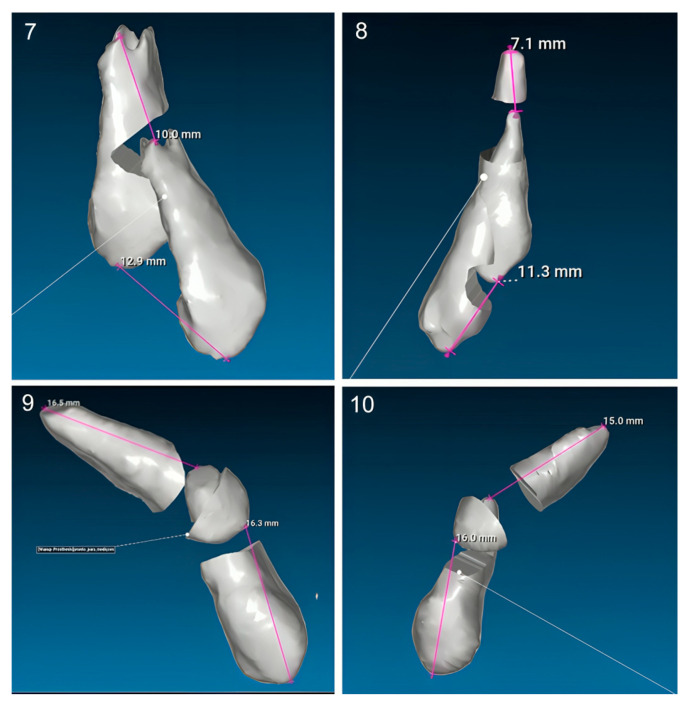
Measurements of all buccally ICs in the sample (n = 4).

The sample consisted of 10 patients aged between 11 and 15 years old (mean = 13.4; SD = 1.12), of which 7 (70%) were male and 3 (30%) were female. Of the total sample, 3 patients had bilaterally ICs and 7 had unilaterally, resulting in a total of 13 IMCs. Nine (69%) of these canines were palatally impacted (Figure 10), while four (31%) were buccally impacted (Figure 11). All patients were treated with Invisalign^®^ aligners, combined with a traction protocol involving elastics, mini-implants, and sectional wires. A Class I canine relationship was achieved in all cases.

To better illustrate the clinical application of the biomechanics used in this study, one representative case was selected. This example reflects the complexity of the included cases and demonstrates how aligners, in conjunction with auxiliary devices, were applied to achieve controlled and effective traction of an impacted maxillary canine.

### 3.2. Representative Clinical Case

**Figure 12:**
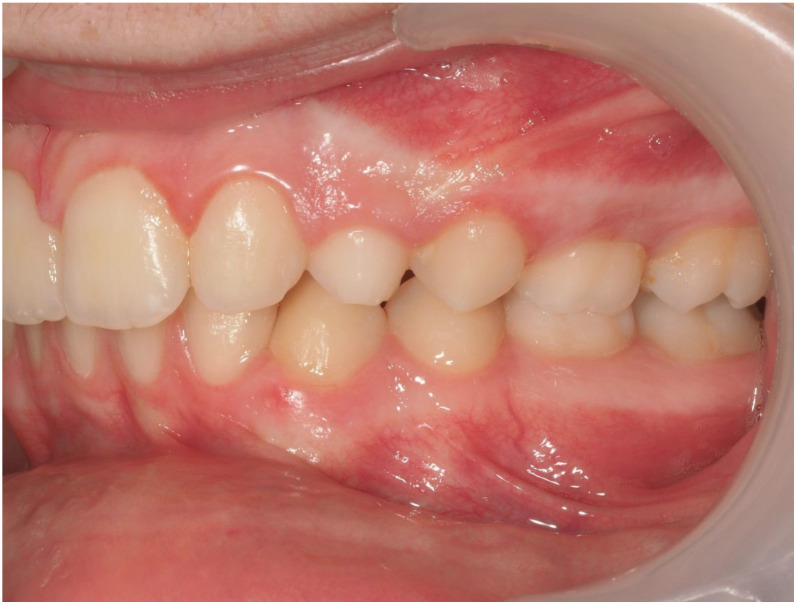
Initial intraoral lateral photo.

**Figure 13:**
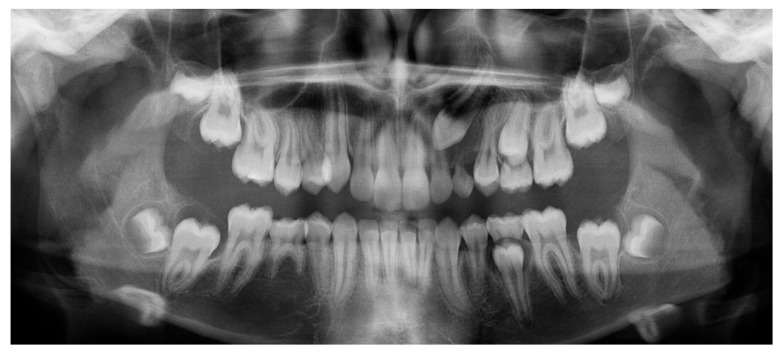
Impacted canine 23 with its cusp palatally positioned to the apical half of the left lateral incisor, with the root significantly curved and inserted into the maxillary sinus.

A 13-year-old male patient presented with Class II Division 2 malocclusion, a bilateral Class II sagittal relationship, and retroclined incisors. Cone-beam computed tomography revealed a palatally impacted maxillary left canine (tooth 23), with its cusp located palatally to the apical half of the left lateral incisor. The root exhibited significant apical curvature, extending toward the floor of the maxillary sinus (Figure 12 and Figure 13), making spontaneous eruption unlikely.

**Figure 14:**
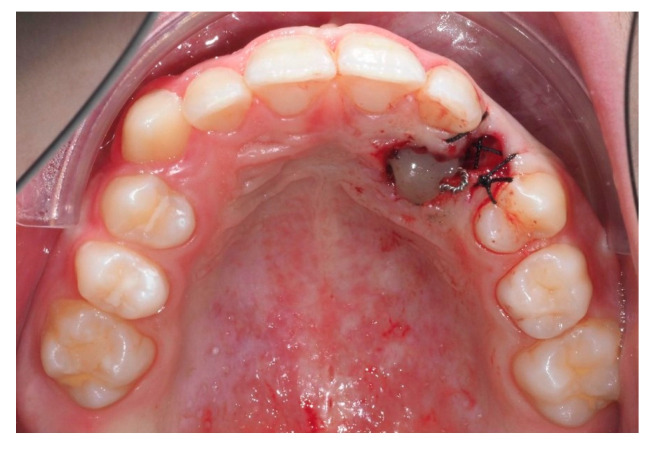
Open surgical exposure of the canine with button fixation and ligature wire for subsequent traction.

An open surgical exposure was performed to access the crown of the IC. A button with a ligature wire was bonded and sealed using two layers of flowable composite resin, enabling controlled traction while minimizing the risk of debonding and simultaneously acting as a protective seal (Figure 14).

**Figure 15:**
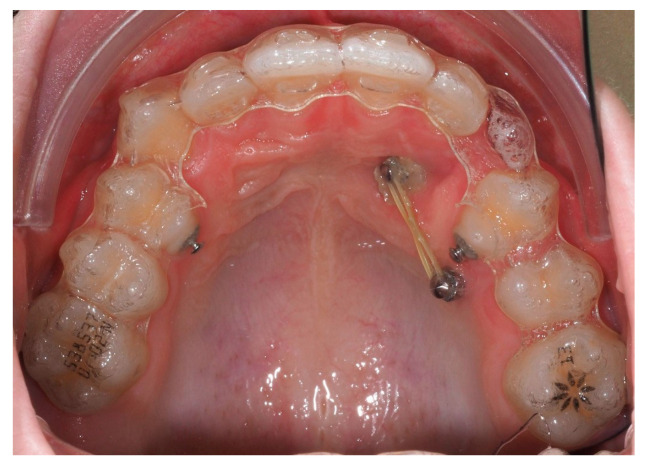
Adjusted aligners, palatal mini-implant, and elastic chain used for distalizing the canine; 7 months after the surgery.

Approximately 7 months later, a palatal mini-implant was placed between teeth 24 and 25, serving as stable anchorage for distal traction using an elastic chain, which was replaced monthly (Figure 15). This movement positioned the canine away from the lateral incisor root. Aligners were customized and adjusted throughout treatment.

**Figure 16:**
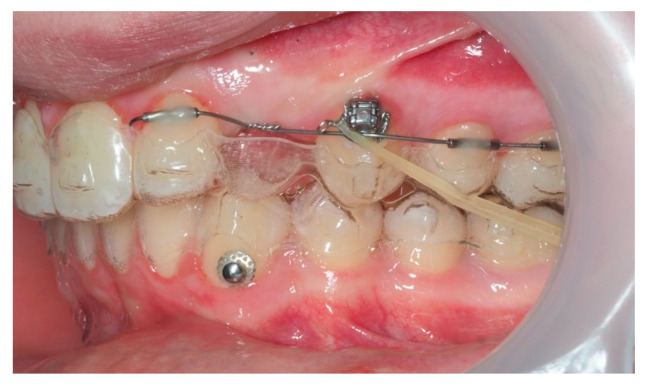
Sectional fixed appliance from teeth 11 to 26, with support for Class II elastics to improve sagittal relationship; 14 months after the surgery.

**Figure 17:**
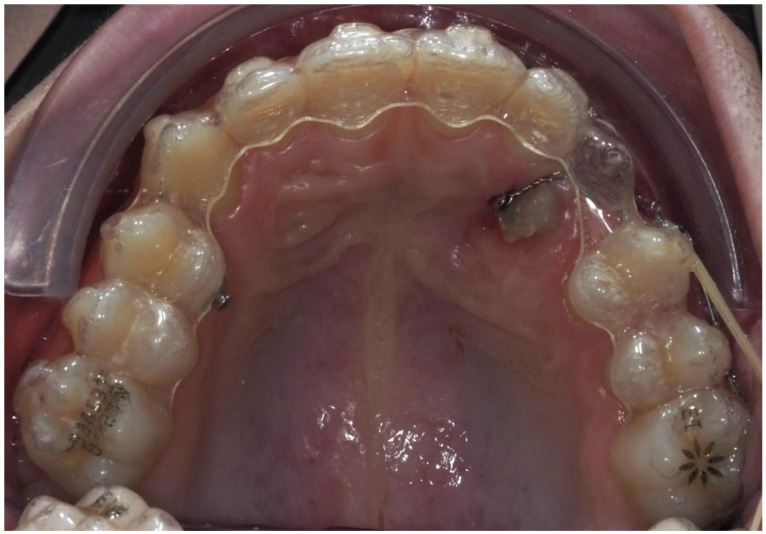
Ligature wire attached to a 0.014” nitinol archwire on the palatal surface of the canine; 14 months after the surgery.

Following initial distalization, a sectional fixed appliance was bonded from tooth 11 to 26. On tooth 24, a bracket with a metallic ligature was used to support Class II elastics. Mini-tubes were bonded on teeth 22, 25, and 26, and the aligners were trimmed accordingly (Figure 16). A ligature wire connected the palatal button on the canine to a 0.014” nitinol archwire (Figure 17).

**Figure 18:**
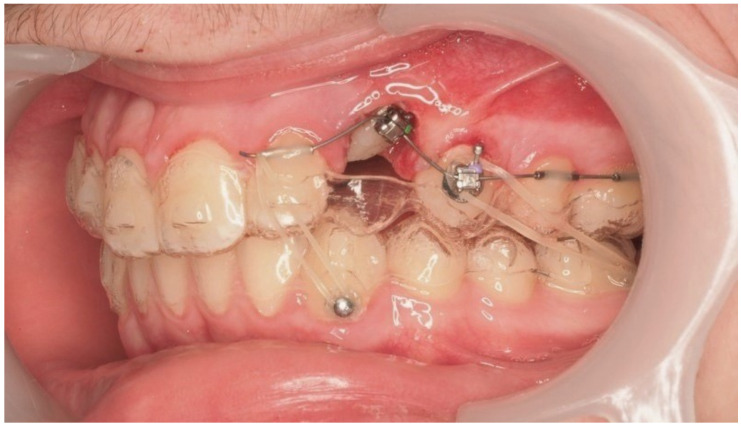
Vestibular traction of the canine, gingival removal, and elastic use for vertical stabilization; 14 months after the surgery.

During vestibular traction, gingival removal was required. The canine presented mesial tipping and rotation. In the 14th month after the surgery, a bracket was bonded to tooth 23, following its inclination. A button was bonded on tooth 33 and connected by elastic to tooth 22 to stabilize the vertical position. Another elastic with a Class II vector was attached from the bracket on tooth 24 to a precision cut in the lower aligner (Figure 18).

**Figure 19:**
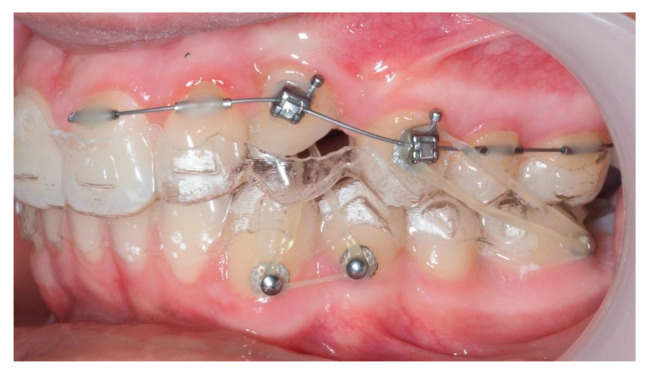
Extension of the sectional fixed appliance up to tooth 21, with an additional tube placed on this tooth; 18 months of treatment.

**Figure 20:**
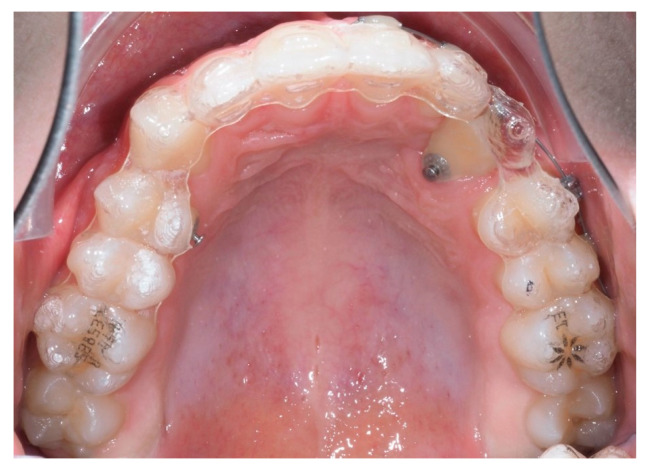
Palatal button on tooth 23 for cross elastic to assist in its vestibularization; 18 months of treatment.

**Figure 21:**
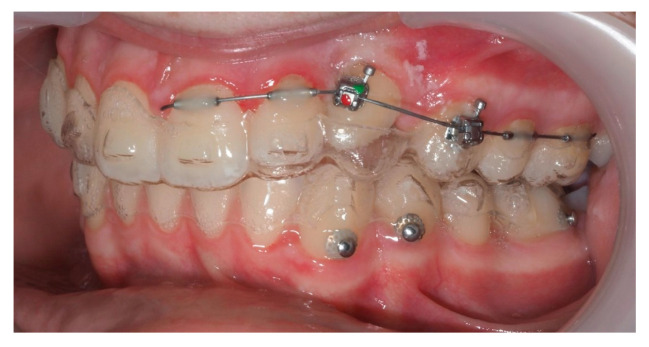
Progressive repositioning of the bracket to aid in extrusion and derotation of the canine; 20 months of treatment.

**Figure 22:**
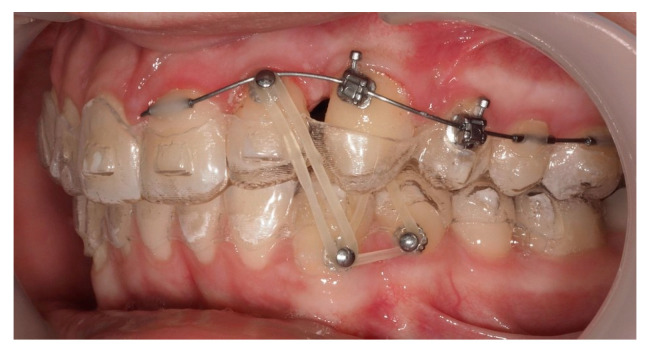
Final adjustments with a button on tooth 22 for vertical recovery and cross elastics for stabilization; 24 months of treatment.

As traction progressed, the sectional appliance was extended to tooth 21 (Figure 19). A palatal button was bonded on tooth 23 to facilitate cross elastic application for vestibular movement (Figure 20). At 20 months, the bracket on tooth 23 was progressively repositioned more cervically and mesially to aid extrusion and derotation (Figure 21). A button was bonded on tooth 22 to support a wire for additional vertical anchorage. Cross elastics were also used between the palatal side of tooth 23 and buttons on teeth 33 and 34, while another elastic connected tooth 22 to 33 (Figure 22).

**Figure 23:**
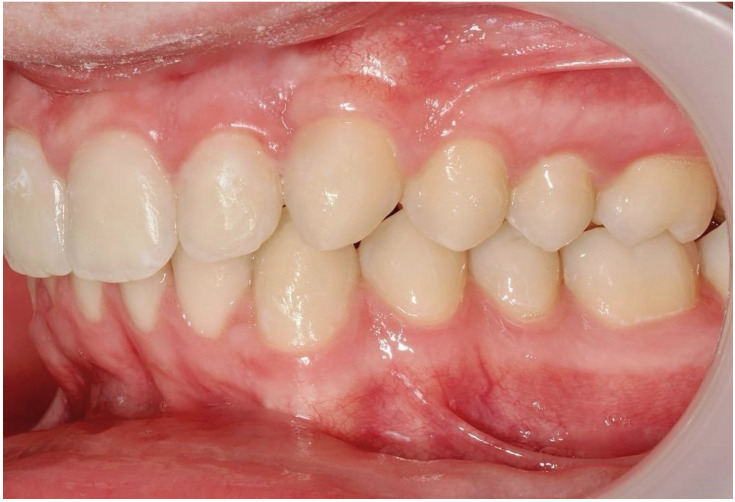
Canine 23 properly positioned in the arch in occlusion; 30 months of treatment.

Ultimately, after 30 months of treatment, canine 23 was successfully repositioned into the dental arch, achieving functional occlusion (Figure 23).

### 3.3. Difficulty Level of Impacted Maxillary Canines Based on CBCT Index

According to the difficulty index proposed by Chauhan et al. [19], 12 IMCs were classified as having maximum difficulty (score 11–15), and 1 IMCs were classified as moderate difficulty (score 6–10). No cases were classified as minimum difficulty (score = 5). The results are presented in Table 1.

The case classified as moderate difficulty received a score of 10, placing it at the upper limit of the moderate category and very close to the threshold for maximum difficulty. The canine was positioned high in the alveolar bone, in close proximity to the lateral incisor root, and located near the buccal cortical plate. Despite the moderate classification by the index, the required orthodontic traction was substantial due to the tooth’s unfavorable position.

The scoring criteria for each factor are listed below [19]:

- Angulation (angle between the long axis of the IC and the mid-sagittal plane): Less than 30° = 1 point. Between 30° and 45° = 2 points. More than 45° = 3 points.
- Vertical position (level of the cuspid tip in relation to the adjacent erupted incisor): At the level of the cementoenamel junction = 1 point. At the middle third of the root = 2 points. At the apical third of the root = 3 points. Above the apical third = 4 points.
- Bucco-palatal position (in axial view, in relation to the ideal final position): Buccal to the final position = 1 point. Palatal to the final position, ≤3 mm = 1 point. Deep palatal position, >3 mm from final position = 2 points.
- Horizontal position (in the transverse plane, relative to adjacent erupted teeth): Overlapping up to half the width of the adjacent tooth = 1 point. Overlapping more than half = 2 points. Completely overlapping the adjacent tooth = 3 points. Completely overlapping and extending beyond the next erupted tooth = 4 points.
- Rotation (labio-lingual orientation of the impacted tooth): Proper orientation (no rotation) = 1 point. Improper orientation (rotation present) = 2 points.

### 3.4. Analysis of the Effectiveness of the Impacted Canine Movement

Using the STL method, the mean tip displacement was 15.37 ± 2.25 mm, and the mean apex displacement was 11.27 ± 3.16 mm. The corresponding displacement speeds were 0.51 ± 0.12 mm/month for the tip and 0.38 ± 0.14 mm/month for the apex. For the CBCT method, the mean tip displacement was 16.40 ± 3.13 mm, and the apex displacement was 9.64 ± 2.92 mm. Tip and apex displacement speeds were 0.48 ± 0.10 mm/month and 0.29 ± 0.10 mm/month, respectively (Table 2).

No statistically significant differences were found between the STL and CBCT methods for either tip displacement (*p* = 0.226) or apex displacement (*p* = 0.363). While the CBCT method recorded slightly higher tip displacement values (16.44 mm vs. 15.38 mm) and lower apex displacement (9.68 mm vs. 10.28 mm), these variations were minimal and clinically insignificant. The effect sizes were small to moderate, with Cohen’s d = 0.469 for tip displacement and Cohen’s d = 0.344 for apex displacement (Table 3).

All 13 IMC cases have an initial CBCT and intraoral scans, but only 8 have a final CBCT. Therefore, for the CBCT method, which requires both CBCT scans, measurements are only possible in 8 cases. All 13 cases are suitable for evaluation using the STL method, as they have the initial CBCT and intraoral scans.

These findings indicate that the STL method provides similar accuracy to CBCT in monitoring the three-dimensional movement of ICs during orthodontic traction, with minor variations that are not clinically significant.

## 4. Discussion

Traditionally, fixed appliances have been the preferred method for performing orthodontic traction of impacted teeth, often in combination with auxiliaries such as a transpalatal arch (TPA). One of their key advantages is the ability to attach the traction accessory directly from the IC to the archwire [21].

A clear advantage of aligners over fixed braces is the ease of maintaining dental hygiene throughout the treatment. This approach has the primary advantage of facilitating oral hygiene, reducing the risk of cavities, inflammatory periodontal diseases, and dental decalcification [22,23]. Moreover, in aligner-based treatment, forces are digitally controlled and evenly distributed across multiple teeth. Sequential tooth movement improves biomechanical precision and limits undesired forces on adjacent structures. The full-coverage design also provides reciprocal anchorage, enhancing the predictability of canine traction. Unlike fixed appliances, aligners eliminate the risk of bracket debonding. However, managing ICs with aligners can be challenging without auxiliary anchorage such as mini-implants [24]. When indicated, their integration enhances the effectiveness of traction. The choice of technique should consider the canine position and anchorage needs [25]. Additionally, aligners may reduce the risk of root resorption and damage to adjacent teeth compared to fixed appliances [26].

In the present study, after a 3D evaluation, all included cases were found to be clinically complex (Table 1), often involving considerable initial distances between the crown and apex, which reflected severe displacement and root angulation. These conditions demanded precise control of movement and stable anchorage. Thus, auxiliary devices such as mini-implants, sectional wires, buttons, and elastics were essential to enable controlled traction and prevent adverse effects on adjacent structures. Furthermore, in cases with CBCT at the end of treatment, no alterations were observed in adjacent teeth, supporting the safety of this approach.

One representative case, illustrated through clinical images (Figure 12, Figure 13, Figure 14, Figure 15, Figure 16, Figure 17, Figure 18, Figure 19, Figure 20, Figure 21, Figure 22 and Figure 23), was selected to exemplify the biomechanical strategy adopted. In this particular case, as in most palatally ICs, anchorage was provided by a palatal mini-implant. Specifically, it was positioned between the first and second premolars, whereas in the other cases, it was placed between the second premolar and the first molar. This positioning allowed for both distalization and vertical traction. In contrast, for buccally ICs, mini-screws were placed either in the infrazygomatic crest or in the lower arch, depending on the desired force vector. Despite the severity of the initial impactions, a Class I canine position was consistently achieved, reinforcing the clinical effectiveness of the proposed biomechanical approach.

Generally, aligners exert pushing forces, while ICs require traction. Since aligners lack elements capable of pulling teeth from ectopic positions, their use in such complex scenarios depends heavily on complementary mechanics. In cases of palatally ICs, mini-implants, and sectional wires allow the application of controlled forces, minimizing collateral movement and reducing risks of root contact or resorption [21].

The high discrepancy between the initial crown and root positions in many cases illustrates the complexity of three-dimensional repositioning required in this type of treatment. Despite this, the controlled and gradual displacement observed supports the effectiveness of the biomechanical strategy employed.

Mampieri et al. described favorable aesthetic outcomes using aligners combined with elastics for IC traction [24]. While this is achievable in cases with favorable canine positions, in our study, spontaneous eruption or exclusive aligner-based traction was not viable. Surgical exposure and multi-phase mechanics were indispensable to guide eruption safely.

Since STL files originate from intraoral surface scans, they cannot visualize internal anatomical structures such as the root apex. While the apical position might seem favorable, this method does not allow confirmation of contact with adjacent structures or assessment of the risk of root resorption. The integration of STL and CBCT data allowed for detailed 3D visualization of the canine’s trajectory, even in severely displaced cases. The overlay of pre- and post-treatment images provided not only quantitative data but also clinical confirmation of successful movement. Given the anatomical challenges, the accuracy of this method reinforces its utility in longitudinal monitoring.

Previous studies comparing TPA and mini-implants have shown no clear superiority in movement speed but highlighted the side effects of conventional anchorages, such as molar tipping [27]. In our protocol, mini-implants provided stable anchorage without significant unwanted effects, even under prolonged traction times, which averaged over 30 months. This further underscores the need for personalized mechanics in complex IC cases.

The average treatment time of about 30 months reflects the complexity of severe impacted canine cases. This longer duration allows for careful, gradual tooth movement in three dimensions, minimizing risk to adjacent structures. Even after the canine is positioned in the arch, treatment may continue with active aligners for minor adjustments to refine and stabilize results before placing the final Vivera retainer. This approach prioritizes biological safety and long-term stability over speed.

Moreover, rapid displacement is not necessarily desirable. Greater movement speed has been linked to an increased risk of damage to adjacent teeth and periodontal tissues. Although rapid displacement may seem efficient, it is not necessarily desirable, as studies have linked higher movement speeds to an increased risk of damage to adjacent teeth and periodontal tissues. Disimpaction of the IC before tooth alignment may help minimize root resorption [28]. Additionally, mini-implants can direct initial traction distally, away from the lateral incisor root, before guiding the IC into the arch. This staged approach minimizes damage risk and improves control over tooth movement.

This staged approach reduces the likelihood of damaging adjacent structures and improves overall control of tooth movement. In cases like those included here, gradual and biomechanically controlled movement was prioritized over speed, emphasizing safety and predictability.

Although rapid displacement may seem efficient, it is not necessarily desirable, as studies have linked higher movement speeds to an increased risk of damage to adjacent teeth and periodontal tissues. In particular, gradual and biomechanically controlled movement, such as disimpaction of the IC before alignment, may reduce the likelihood of root resorption and other complications, thus prioritizing safety and long-term predictability over speed.

The use of STL scans overlaid on initial CBCTs proved to be a clinically reliable method for assessing tooth displacement when the crown had erupted. Although CBCT remains essential for initial diagnosis and planning, this hybrid method offers a viable low-radiation option for end-of-treatment assessment in complex cases.

Overall, our findings support the use of aligners combined with well-designed auxiliaries as a potentially effective and safe strategy for treating severe ICs. Even in cases involving large initial displacements and difficult positions, the outcomes demonstrate that complex movements can be achieved with precision. In the cases evaluated with final CBCT, no adverse effects, such as root resorption or damage to adjacent structures, were observed.

## 5. Conclusions

The orthodontic traction of impacted maxillary canines, particularly in complex cases with significant crown–root displacement and unfavorable positioning, can be successfully achieved through the combined use of aligners, elastics, mini-implants, and sectional wires. This integrative approach proved biomechanically effective and clinically safe and with no adverse effects detected on adjacent teeth throughout the treatment period in the cases assessed via CBCT.

Intraoral scans (STL) are a reliable tool for tracking canine movement once the crown has erupted. When overlaid with the initial CBCT, STL data enabled an accurate evaluation of the final canine position, both crown and root, eliminating the need for a second CBCT and reducing overall radiation exposure. This method is applicable only when the canine crown has erupted and captured by the intraoral scanner, ensuring sufficient exposure for accurate overlay and evaluation.

This strategy provides a precise, low-radiation method for follow-up once canine eruption is confirmed while emphasizing the essential role of CBCT at the diagnostic stage for safe, individualized treatment planning in complex impactions. Although CBCT provides valuable information regarding root position, potential contacts, and risk assessment, STL scans offer a reliable, radiation-free alternative for monitoring crown movement during treatment. In selected cases, STL-based monitoring can reduce or even replace the need for repeated CBCT scans, particularly during the active treatment phase, without compromising clinical safety or precision.

## Figures and Tables

**Table 1:** Difficulty level of impacted maxillary canines based on CBCT index.

Sample	Angulation	Vertical Position	Bucco-Palatal Position	Horizontal Position	Rotation	Total
Case 1	3	3	2	2	2	**12**
Case 2 A	2	2	2	4	2	**12**
Case 2 B	3	2	2	2	2	**11**
Case 3 A	2	2	2	4	2	**12**
Case 3 B	2	2	2	4	2	**12**
Case 4	2	3	2	4	2	**13**
Case 5 A	3	2	2	4	2	**13**
Case 5 B	3	2	2	3	2	**12**
Case 6	3	2	2	3	2	**12**
Case 7	3	2	1	3	2	**11**
Case 8	3	2	1	3	2	**11**
Case 9	3	3	1	2	1	**10**
Case 10	3	4	1	1	2	**11**

Difficulty: Minimum (5), Moderate (6–10), Maximum (11–15).

**Table 2:** Three-dimensional measurements and displacement speed of the IC in the STL method and CBCT method.

Method	Measurement	N	Mean ± SD (mm)
CBCT	Tip displacement	8	16.40 ± 3.13
	Apex displacement		9.64 ± 2.92
	Tip displacement speed (mm/month)		0.48 ± 0.10
	Apex displacement speed (mm/month)		0.29 ± 0.10
STL	Tip displacement	13	15.37 ± 2.25
	Apex displacement		11.27 ± 3.16
	Tip displacement speed (mm/month)		0.51 ± 0.12
	Apex displacement speed (mm/month)		0.38 ± 0.14

Abbreviations: CBCT: Cone-Beam Computed Tomography, STL: STL file format.

**Table 3:** Comparison of tip displacement and apex displacement between the two methods.

	N	Mean ± SD (mm)	*p*-Value	Cohen’s d
Tip Displacement Measurement Method				
CBCT	8	16.44 ± 3.13	0.226 *	0.469
STL		15.38 ± 2.86		
Apex Displacement Measurement Method				
CBCT	8	9.68 ± 2.95	0.363 *	0.344
STL		10.28 ± 2.73		

Abbreviations: CBCT: Cone-Beam Computed Tomography, STL: STL file format. * Not statistically significant (*p* ≥ 0.05).

## Data Availability

Data supporting the findings of this study are available from the corresponding author upon reasonable request.

## Abbreviations

The following abbreviations are used in this manuscript:

CBCT	Cone-Beam Computed Tomography
ErC	Erupted Canine
IC	Impacted Canine
IMC	Impacted Maxillary Canines
OPG	Orthopantomography
STL	STL file format
TPA	Transpalatal arch
